# Isolation of cubic Si_3_P_4_ in the form of nanocrystals

**DOI:** 10.3762/bjnano.14.80

**Published:** 2023-09-26

**Authors:** Polina K Nikiforova, Sergei S Bubenov, Vadim B Platonov, Andrey S Kumskov, Nikolay N Kononov, Tatyana A Kuznetsova, Sergey G Dorofeev

**Affiliations:** 1 Department of Chemistry, Lomonosov Moscow State University, 1-3 Leninskie Gory, Moscow, Russia; 2 Shubnikov Institute of Crystallography, Federal Scientific Research Centre «Crystallography and Photonics», Russian Academy of Sciences, 59 Leninskiy prospekt, Moscow, Russia; 3 Prokhorov General Physics Institute, Russian Academy of Sciences, 38 Vavilov Str., Moscow, Russia

**Keywords:** ampoule annealing, defective zinc blende structure, DFT calculations, semiconductor nanocrystals, silicon phosphide

## Abstract

This article describes an approach for synthesizing silicon phosphide nanoparticles with a defective zinc blende structure under mild conditions through thermal annealing of hydrogenated silicon nanoparticles with red phosphorus. The synthesized Si_3_P_4_ nanoparticles were analyzed using FTIR, XRD, electron diffraction, EDX, TEM, Raman spectroscopy, X-ray fluorescence spectrometry, and UV–vis spectrophotometry. For the isolated cubic Si_3_P_4_ phase, a cell parameter of *a* = 5.04 Å was determined, and the bandgap was estimated to be equal to 1.25 eV. Because of the nanoscale dimensions of the obtained Si_3_P_4_ nanoparticles, the product may exhibit several exceptional properties as a precursor for diffusion doping of wafers and as anode material for Li-ion batteries. A similar method with a hydrogenation step offers the possibility to obtain other compounds, such as silicon selenides, arsenides, and sulfides.

## Introduction

Advancements in electronics and related fields are calling for new ways of synthesizing compounds. Subsequently, recognizing and utilizing the special properties of nanoparticles (NPs) of new materials using emerging methods offers a number of potential benefits to both science and industry. These methods often employ new strategies for the synthesis of specific classes of substances. For example, the widely known development of perovskite synthesis through ion exchange reactions has attracted attention in scientific as well as engineering communities [1].

Materials based on compounds of Earth-abundant elements are extensively sought for. With regard to the Si–P system, novel compounds are still discovered, and research on nanoscale forms of silicon phosphides is ongoing. For instance, nanocrystalline cubic SiP was formed as inclusions in Si wafers implanted with P^+^ ions and laser-annealed at temperatures of 450–850 °C [2]. Recently, NPs of elemental compositions close to SiP and SiP_2_ have been observed in thermal annealing products of non-stoichiometric silicon oxide containing high amounts of phosphorus introduced during the fabrication; the resulting composition seems to arise from Si and P supersaturation levels [3–4]. The formation of precipitates in supersaturated silicon has also been considered as a possible mechanism involved in the deactivation of dopants [5–7]. To date, Si_3_P_4_ compounds have not been experimentally synthesized either under atmospheric or high pressure. Since pseudocubic Si_3_P_4_ is bound to be metastable according to the phase diagram [8], any method of its synthesis must involve out-of-equilibrium conditions. The majority of theoretical studies on numerous Si_3_P_4_ phases have shown pseudocubic (tetragonal) Si_3_P_4_ to be energetically favored [9–14]. The calculated lattice constant *a* and the ratio *c*/*a* lie within the ranges of 4.961–5.093 Å and 0.994–1.003, respectively.

Among the properties of silicon-based materials, one may note a high specific capacity, which is crucial for Li-ion batteries. For the practical application of the materials, however, the problems of low conductivity and dramatic volume expansion of Si after full lithiation must still be solved [15]. To this end, silicon phosphides are actively studied. Layered silicon phosphide and diphosphide, for example, provide facile lithium transport and possess a promising capacity of 2700 and 862/737 mA·h·g^−1^, respectively [16–17]. In the case of the synthesized nanoscale particles, a high level of Li mobility can be reached because of their developed surface and the interconnected ordered network of silicon vacancies with respect to the zinc blende structure. The expansion problem, however, is likely to be mitigated by the presence of interparticular voids, making the synthesized cubic Si_3_P_4_ nanocrystallites a prospective material for Li-ion batteries. Additionally, NPs may prove promising in the preparation of microcircuits through the introduction of P donors by diffusion doping of wafers with an efficiency comparable to monolayer doping [18] or ion implantation [19].

This paper outlines the successful formation of the cubic Si_3_P_4_ phase under mild conditions. The technique developed for this investigation requires temperatures as low as 400 °C with no elevated pressure. Prior to this study, bulk cubic sphalerite-type SiP had only been synthesized above 1700 °C under a pressure of 40–50 kbar [20].

## Results and Discussion

Initially, the objective of the experiment was to obtain silicon NPs with a controlled distribution of phosphorus as a dopant. From the outset, phosphorus diffusion through hydrogenated and oxide layers was surmised to be different. In the case of similar previously performed syntheses of Si NPs with an oxide layer [21], phosphorus diffuses deep into the NP cores and distributes rather homogenously with a minor separation occurring on the surface of the NPs [22–23]. The temperature (670 °C) and duration *t* of the preliminary experiment were chosen through the estimation of the diffusion length *l* from bulk silicon diffusion constants [24] using the following equation:


[1] $$l=1.274\sqrt{Dt},$$

where *D* is the diffusion constant under the chosen conditions. Based on a calculated length of 2.4 nm, it was expected that the phosphorus would distribute near the surface of 20 nm Si NPs.

**Figure 1:**
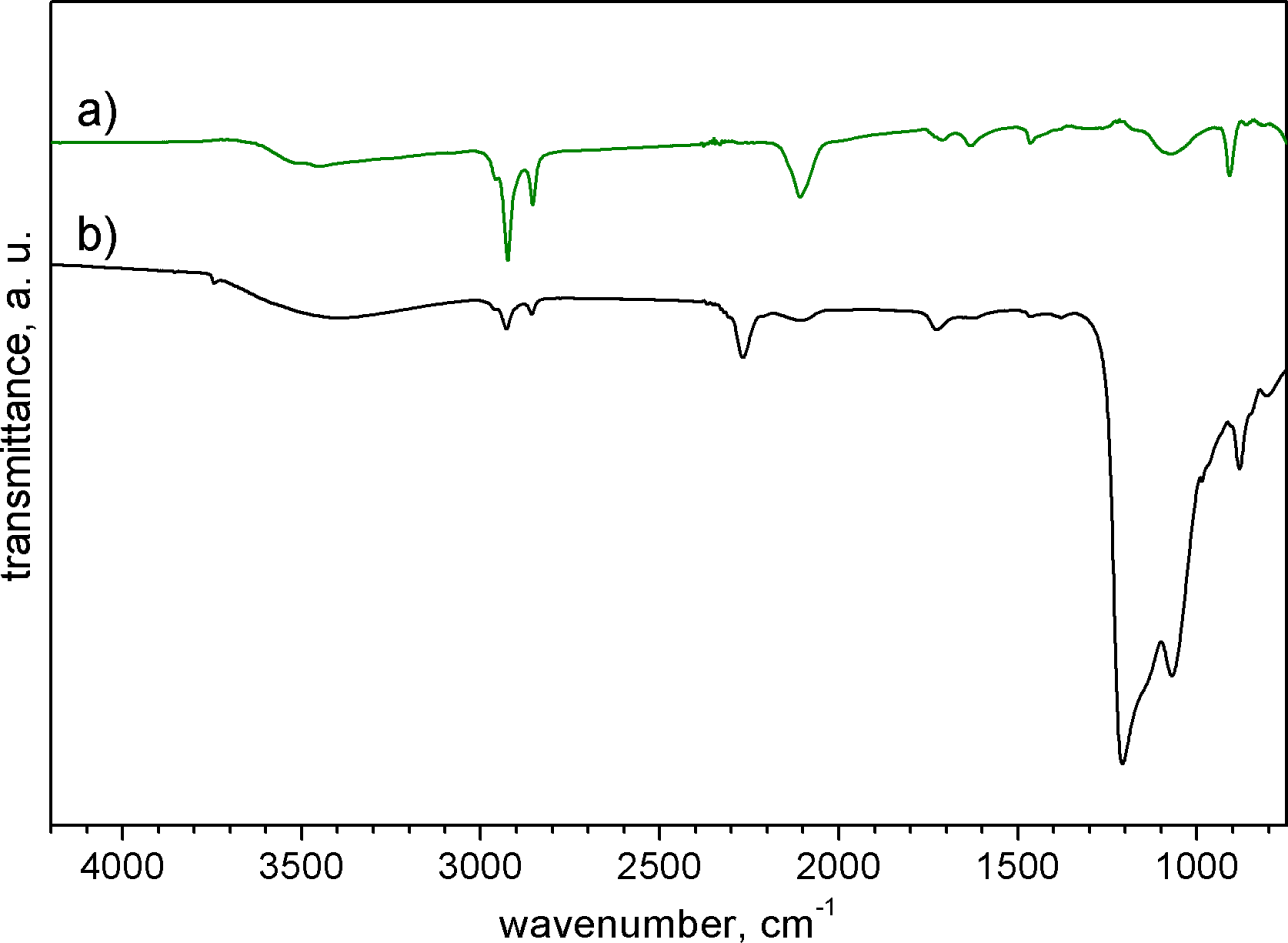
IR spectra of Si NPs (a) upon etching and (b) before etching.

To examine the hypothesis of a controlled surface phosphorus distribution, a hydrogenation of the Si NPs was performed first (the results of the FTIR spectroscopy of the etching product are shown in Figure 1). The bands with the wavenumbers of 2105 and 2900 cm^−1^, and the broad signal at 3400 cm^−1^ were attributed to vibrations of Si–H, C–H, and O–H bonds, respectively [25]. The IR spectra of the unetched Si NPs display bands in a wavenumber range of 1000–1200 cm^−1^, which were associated to the stretching vibrations of Si–O bonds occurring in the oxide layer on the surface of the Si NPs [26–28]. A detailed comparison of bands and vibrations can be found in Table S1 of Supporting Information File 1.

**Figure 2:**
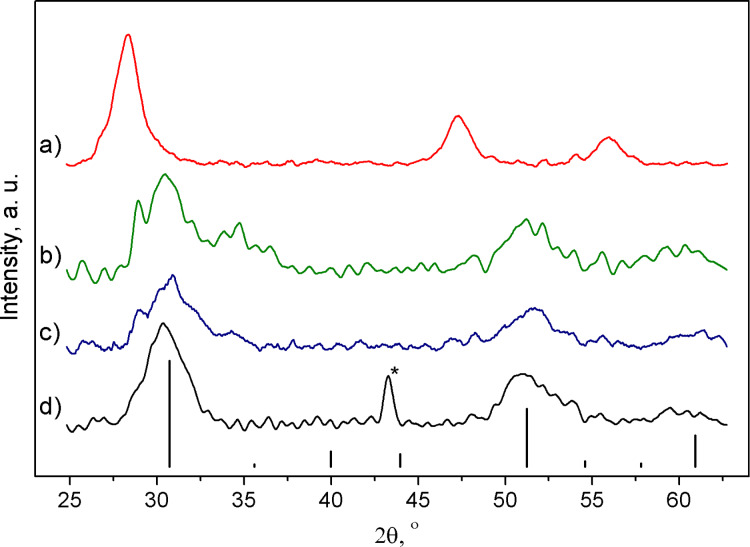
XRD diffraction patterns of (a) Si, (b) SP900, (c) SP670, and (d) SP400. The asterisk denotes a peak from the copper admixture, as discussed in the main text. The vertical lines correspond to the theoretical diffraction pattern of Si_3_P_4_; the vertical lines height is scaled to relative intensity of the respective maximum.

The analysis of the diffractogram of the etched NPs mixed with red phosphorus and annealed at 670 °C (SP670; the other samples have a similar designation, namely “SP” followed by the temperature of the plateau of the annealing. The sample pre-annealed at 900 °C has a postscript “pre-an.”.) showed a significant discrepancy to the diffraction pattern of silicon (Figure 2). In addition, the synthesized NPs disintegrated entirely in nitric acid in contrast to Si NPs. This outcome indirectly confirms the formation of a different compound.

To further ascertain the formation of a new substance, syntheses at temperatures of 400 and 900 °C were conducted. A synthesis at 400 °C was performed in order to observe the influence of hydrogen preservation on the process of formation of the new compound as well as to attempt postsynthetic hydrosilylation to protect the particle cores from air and moisture. A temperature range of 400 to 500 °C was shown to be consistent with Si–H bond cleavage and hydrogen desorption from silicon surfaces [29] (including Si NPs [30]) with the bond completely disappearing at 700 °C as indicated by FTIR spectra [31–33]. A temperature of 900 °C was chosen to assess the probability of formation of another Si*_x_*P*_y_* phase as cubic Si_3_P_4_ is metastable in bulk [8].

In Figure 2, each annealed sample has three peaks with values of 2θ equal to 30.7°, 51.3°, and 60.9°; sample SP900 exhibited an additional fourth peak at an angle of 34.6°. The former three reflexes can readily be indexed as, respectively, (111), (220), and (311) reflections of a cubic lattice with a cell parameter of 5.04 Å. An ICDD database search uncovered a cubic sphalerite type SiP phase with a lattice constant of 5.241 Å; the substantial difference of the observed value confirms formation of a new substance [20]. According to DFT calculations in the GGA approximation, a pseudocubic defective zinc blende Si_3_P_4_ phase was predicted to possess lattice constants *a* = 5.038 Å and *c* = 5.038 Å consistent with our experimental results [11]. Given that the values of the parameters *a* and *c* cannot be experimentally discerned, the lattice was subsequently treated as a cubic one belonging to the 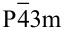 space group. The theoretical diffraction pattern for cubic Si_3_P_4_ with a lattice parameter of 5.04 Å and bond length of 2.27 Å (superimposed in Figure 2) follows the experimental diffractograms in terms of relative intensities. Three prominent maxima were observed, while other signals, weaker than the (311) reflex by at least a factor of two, were ill-defined because of an overlap of the broadened reflections and a low signal-to-noise ratio. As no systematic effect of the synthesis temperature on the cell parameter was observed (Table 1), all samples were expected to conform to the elemental composition Si_3_P_4_. The emergence of an additional diffraction maximum for the sample SP900 was likely due to partial degradation of the compound. Signs of decomposition could be seen in the diffraction pattern of sample SP670 as well, albeit to a lesser extent.

**Table 1:** Synthesis conditions and results of X-ray fluorescence analyses and XRD analyses of samples annealed with phosphorus at 670, 400, and 900 °C.

Sample	Temperature, °C	Duration, hours	Stoichiometry Si/P	Cell parameter (*a*), Å

SP670	670	72	1:1.082	5.01
SP670 pre-an.	670	72	1.644:1	5.04
SP550	550	96	1.366:1	5.05^a^
SP400	400	336	1.279:1	5.08
SP900	900	2	1.142:1	5.04

^a^The cell parameter of SP550 was estimated from electron diffraction experiments.

**Figure 3:**
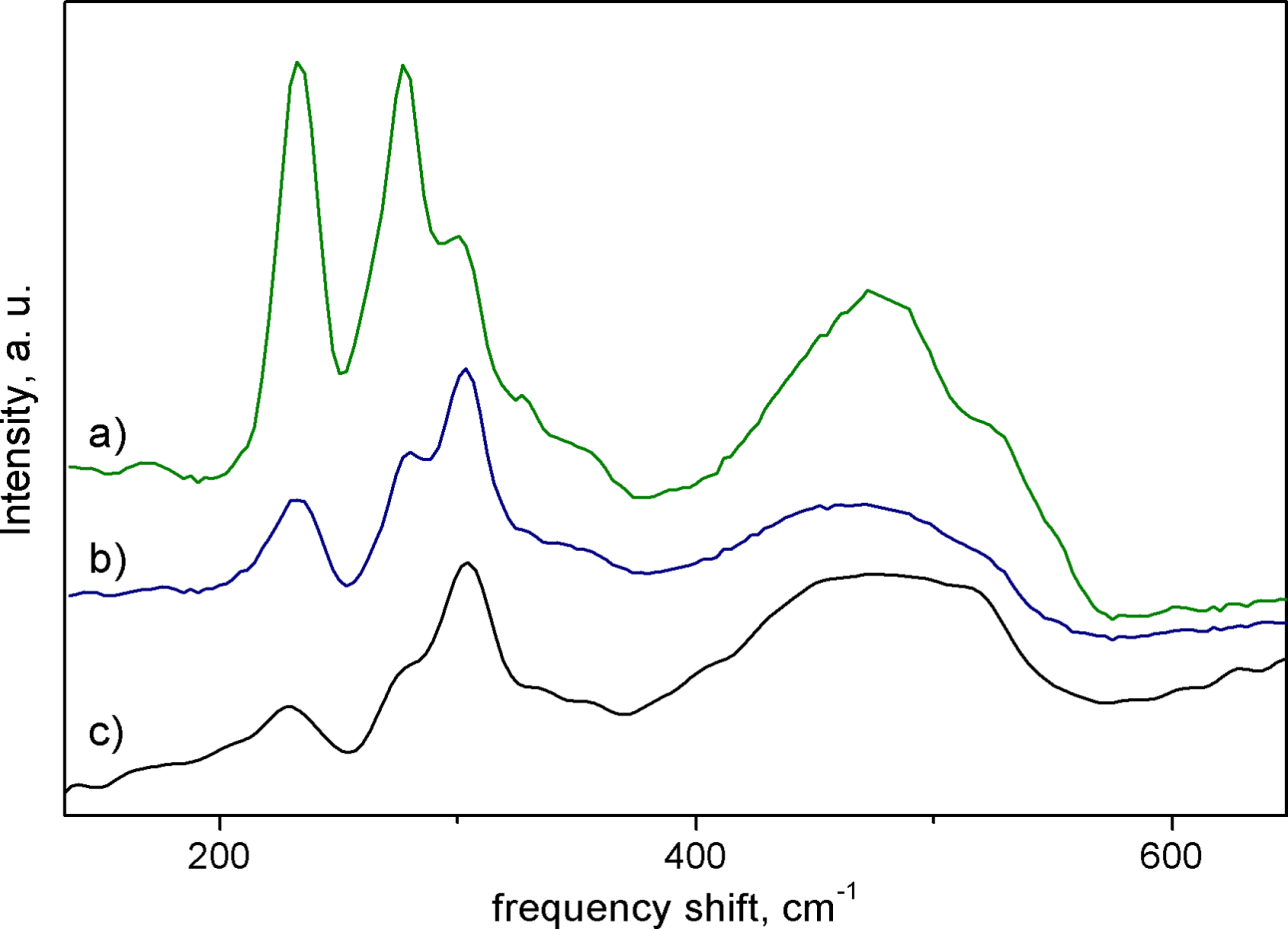
Raman scattering spectra of (a) SP900, (b) SP670, and (c) SP550.

Raman spectra of the samples SP550, SP670, and SP900 are shown in Figure 3 (SP400 exhibited photoluminescence of an organic origin that hindered Raman studies). The spectra consist of multiple bands that provide a stark contrast to the zinc blende SiP structure, which exhibits a singular Raman mode (with a LO–TO splitting as the Si–P bond is slightly polar). As the relative prominence of the bands at 233 and 279 cm^−1^ was enhanced in the case of sample SP900, they were attributed to a decomposition product of Si_3_P_4_. A number of other features were observed including a sharp maximum at 303 cm^−1^, a discernible shoulder at ca. 350 cm^−1^, a weak band centered at 170 cm^−1^, as well as a broad band centered at 475 cm^−1^ with a shoulder at ca. 525 cm^−1^. The band at a wavenumber of 525 cm^−1^ can plausibly be designated to the highest-frequency Si–P vibration as the Γ-point optical phonon of Si is positioned at 520.8 cm^−1^. Interestingly, the observed Raman spectrum for SP900 is very similar to that of the product of silicon and phosphorus MBE co-deposition at 3:2 molar ratio [34]. This substance was reported as amorphous; however, it could contain Si_3_P_4_ NPs along with other Si*_x_*P*_y_* compounds.

Factor group analysis for the 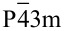 Si_3_P_4_ unit cell yields Γ_optic_ = A_1_ + E + 2T_1_ + 3T_2_, Raman active vibrations of the representations A_1_, as well as E and T_2_, totaling five prior to splitting. DFT calculations were used to tentatively assign symmetries to the observed Raman modes (Table 2). The resultant structural parameters were reasonably close to that of [11] with a lattice parameter of 5.054 Å and bond length of 2.27 Å. The fully symmetric A_1_ vibration frequency was precisely determined in our calculations, while the others were off by up to ca. 34 cm^−1^ with respect to the experimental values. This discrepancy was credited to a significant contribution of van der Waals interactions in the studied structure (The P–P distance in the vacancy was 3.22 Å, which is lower than the doubled van der Waals radius of phosphorus [35]).

**Table 2:** Calculated frequencies of optical phonons (cm^−1^) and preliminary designation of the experimentally observed vibrational frequencies of Si_3_P_4_ NPs.

	Theoretical	Experimental		Theoretical	Experimental

T_1_	156	silent	T_2_ (TO)	412	not observed
T_2_ (TO)	183	170	T_2_ (LO)	420	
T_2_ (LO)	185		T_1_	447	silent
A_1_	302	303	T_2_ (TO)	510	494
E	384	ca. 350	T_2_ (LO)	522	525

Slight shifts of frequencies are not uncommon for DFT calculations in such cases even when appropriate corrections are made [34]. The broad signal at 475 cm^−1^, likely corresponding to an amorphous admixture, obscures two high-frequency T_2_ modes (the position of one of them will be addressed in the IR spectra discussion below). Fundamentally, the consistency between the experiment and computations can be considered satisfactory. Reports on the fabrication of 3:4 binary compounds with defective zinc blende structure are scarce [36–37]; to the best of the authors’ knowledge, a comparison of calculated vibrational spectra with experimental ones has never been undertaken.

**Figure 4:**
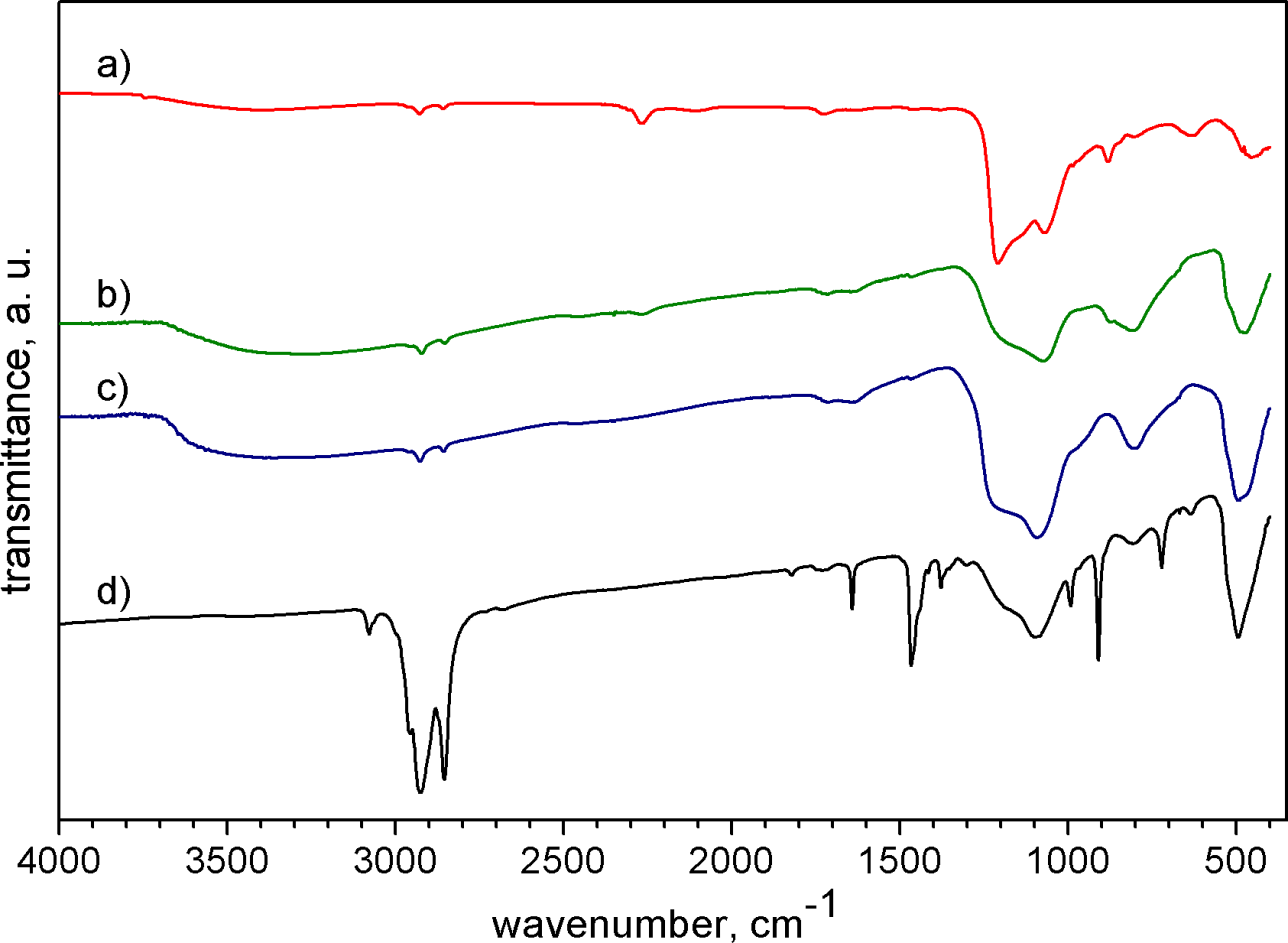
IR spectra of (a) Si, (b) SP900, (c) SP670, and (d) SP400.

IR spectra of the annealed samples revealed a pronounced absorption band with a maximum at 490–494 cm^−1^ and a shoulder at ca. 525 cm^−1^, which is consistent with the earlier assignment of the IR-active T_2_ mode to the highest-frequency vibration (Figure 4). The synthesized Si_3_P_4_ NPs were oxidized on the surface during exposure to air, which produced a signal in the wavenumber range of 1130–1100 cm^−1^. Because of the hydrosylilation step for the sample SP400, its spectrum features prominent signals of organic fragments (Supporting Information File 1, Table S1). Nevertheless, with the absorption of Si–O–Si stretching at 1186 and 1098 cm^−1^, it can be concluded that Si_3_P_4_ synthesized at 400 °C degrades when stored in air. Accordingly, the hydrosylilation of the NPs with octadecene occurred only partially.

It is worth noting that promising results were obtained by treating freshly synthesized Si_3_P_4_ NPs with 1-dodecanol. The rationale behind such functionalization is that fatty alcohols will form ether bonds with surface silicon atoms upon alcoholysis, and bulky alyphatic chains will provide a steric barrier and deter further surface reactions. In this example, the obtained sols showed no signs of particle agglomeration for at least a month.

**Figure 5:**
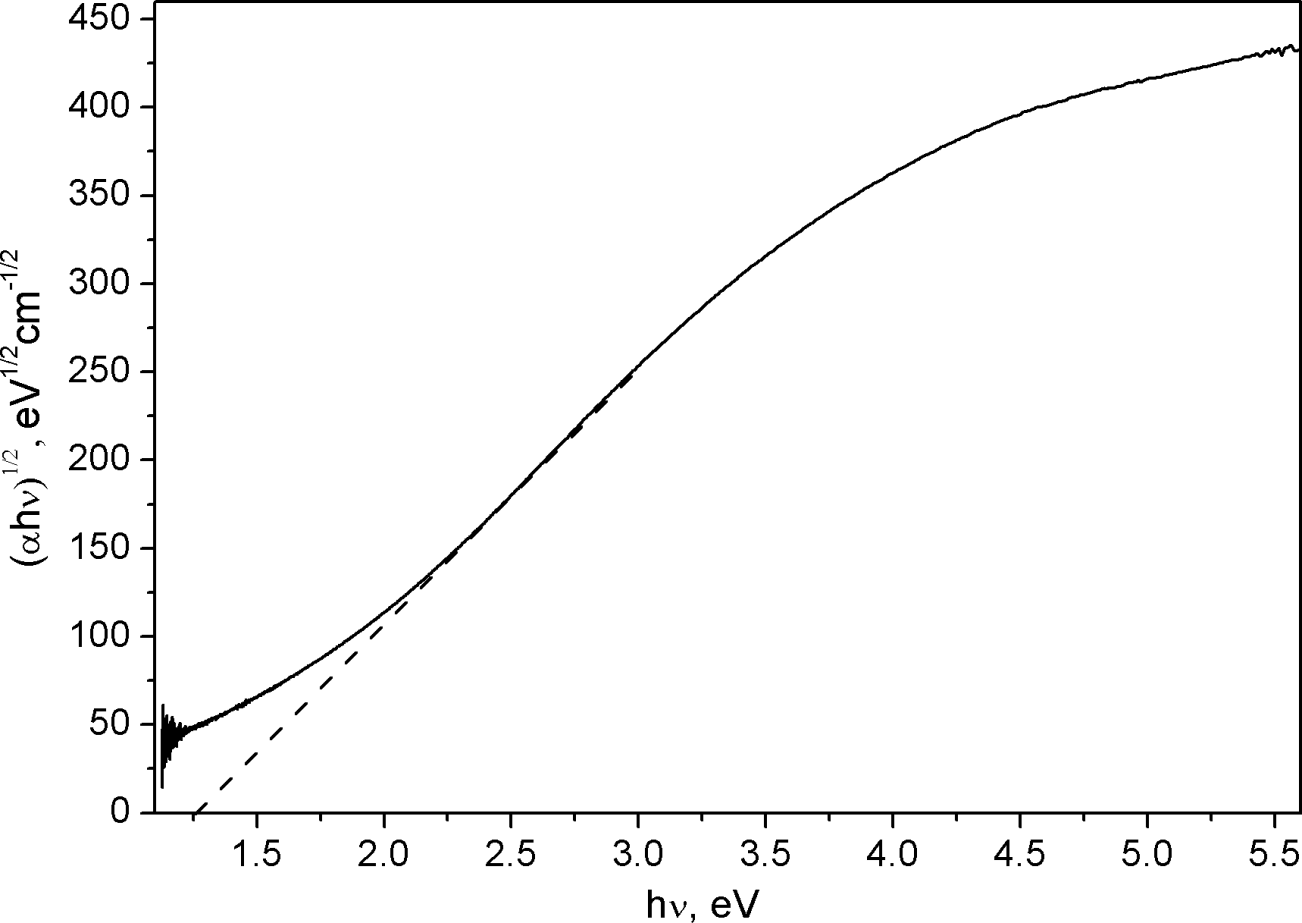
Tauc plot of the optical absorption of a Si_3_P_4_ NPs sample.

The synthesized samples were brown powders that formed brown sols in acetonitrile, quite similar in appearance to those of Si NPs. Upon examination of the UV–vis absorption spectra, a bandgap of 1.25 eV was established using Tauc plot (Figure 5). A simpler approach for a disordered semiconductor material would be to find the energy at which the attenuation coefficient exceeds 10^4^ cm^−1^ (the optical gap estimated this way amounts to 2.3 eV). All of these observations disprove earlier theoretical assumptions of defective zinc blende Si_3_P_4_ as being a narrow-bandgap semiconductor [11]. Notwithstanding, the theoretically predicted maximum at 4 eV was appropriately reproduced in the absorption spectrum.

The samples synthesized at 400, 670, and 900 °C including SP670 pre-an. have a similar crystal structure; they differ, however, in the Si/P elemental ratio. This phenomenon might be induced by a reaction of Si_3_P_4_ NPs with the air moisture resulting in a fluctuating ratio of SiO_2_/Si_3_P_4_ in the particles. The possibility of phosphorus sorption on the well-developed surface of NPs further complicates the determination of the ratio.

**Figure 6:**
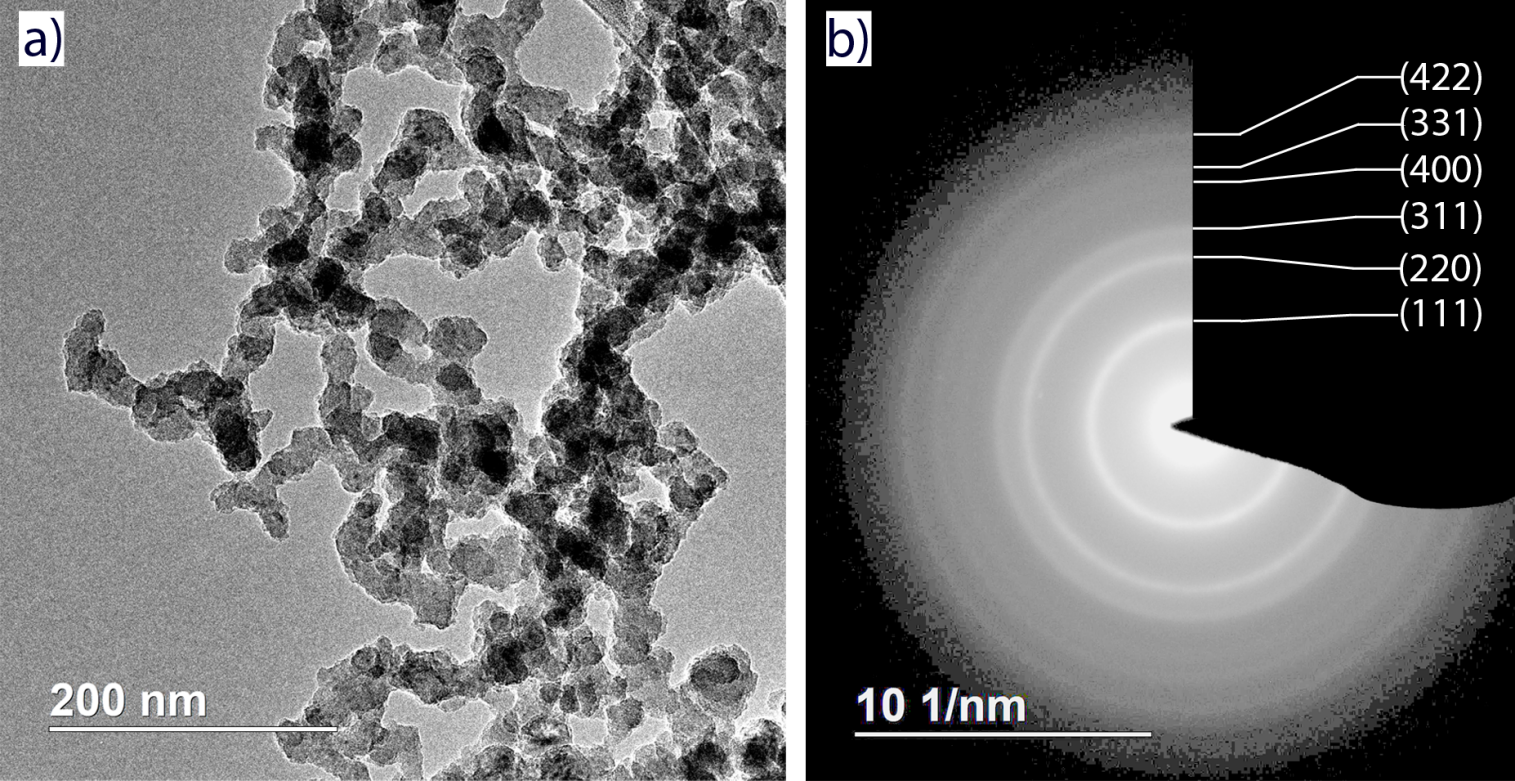
(a) Bright-field TEM image and (b) electron diffraction of the sample SP550.

As higher temperatures were found to promote Si_3_P_4_ decomposition, sample SP670 as well as the freshly prepared sample SP550 were selected for the TEM studies. A typical TEM bright-field image is shown in Figure 6a. The particles show a profound tendency to agglomerate, not unlike the parent oxidized Si NPs [38]. The agglomeration obstructs size determination; thus, the linear dimensions of the particles could only be estimated to be in the range of 10–50 nm.

**Figure 7:**
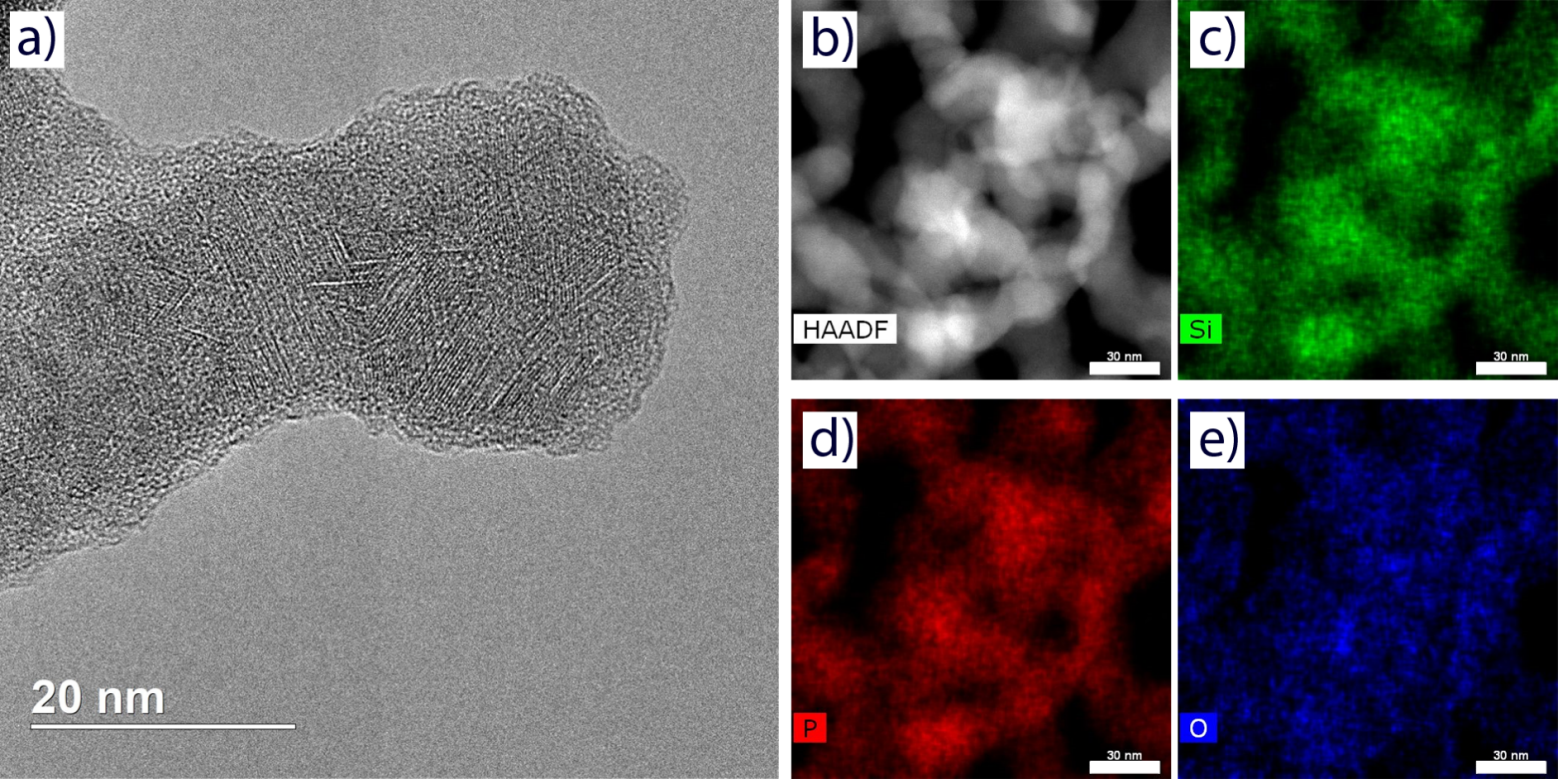
TEM studies of the sample SP550. (a) High-resolution TEM image; (b) HAADF-STEM image; EDX elemental maps for (c) Si Kα, (d) P Kα, and (e) O Kα. The scale bar in images (b–e) is 30 nm.

The particles are polycrystalline and highly defective as evident from the high-resolution image (Figure 7a). The size of the crystalline domains does not exceed 10 nm in its longest dimension. This is consistent with the average size of crystallites of 4 nm determined from the XRD data using the Scherrer equation. The electron diffraction ring pattern (Figure 6b) corresponds to a cubic lattice with a parameter of 5.05 Å (apart from the (111), (220) and (311) reflections discussed earlier, the less noticeable (400), (331), (422) and (511) signals were observed). Amorphous material is present in the sample, which is most noticeable as a ca. 2 nm layer on the surface of the NPs (Figure 7a).

Energy-dispersive X-ray (EDX) elemental maps (Figure 7c,d) for Si Kα and P Kα do not show any significant variation and closely resemble the HAADF-STEM images of the corresponding sample regions (Figure 7b). The two elements are, thereby, viewed as strongly associated and distributed rather homogeneously in the NPs. When the particle agglomeration is not as severe (border regions of Figure 7e), the O Kα map tends to outline the shape of the NPs, confirming surface hydrolysis and oxidation of the material in air during storage.

When the EDX spectra (Supporting Information File 1, Figure S1) were normalized to the intensity of the Si Kα line, it was observed that the growth in the relative intensity of the O Kα line corresponds to the decrease of the same parameter of the P Kα line. This corroborates the loss of phosphorus (presumably in the form of phosphine) during hydrolysis. Silicon and phosphorus in the samples appeared approximately in equimolar quantities, even with an excess of phosphorus in the less degraded regions of the SP550 sample (by an estimated factor of up to 1.3). When the silicon oxide shell was taken into account, the results of elemental analysis were found compatible with the formation of the Si_3_P_4_ compound.

Regardless of the performed calculations of phosphorus penetration into Si NPs, in which the process was modeled as diffusion doping, samples consisted primarily of the new compound of cubic Si_3_P_4_. This fact revealed that the mechanism of Si_3_P_4_ formation may differ. For example, one might suppose that the amorphous Si layer, present in the nanocrystalline products of CVD synthesis [39], could provide a faster diffusion channel and play a key role in Si_3_P_4_ formation. Moreover, the heating of amorphous silicon is also known to induce an irreversible transition to the crystalline state above 700 °C [40]. Next, a synthesis of pre-annealed SP670 that consisted of vacuum annealing of the Si NPs at 900 °C and subsequent HF etching followed by annealing with red phosphorus at 670 °C was carried out. The successful synthesis of cubic Si_3_P_4_ under these conditions confirmed that the external amorphous silicon was not the defining factor in the formation of the Si_3_P_4_ phase.

Also worth noting was that the synthesis of cubic Si_3_P_4_ NPs could be performed immediately after laser-induced or plasma-enhanced chemical vapor deposition of nanosilicon from the silane precursor (since the NPs obtained this way are readily hydrogenated). Additionally, there is preliminary XRD evidence that SiP NPs are formed when phosphorus is present in less than an equimolar quantity with respect to hydrogenated silicon. As cubic SiP and Si_3_P_4_ are very similar in structure, they are quite likely to be miscible at the nanoscale resulting in a “vacancy doping” scenario with possible control over the width of the bandgap through selection of the composition.

## Conclusion

A synthesis of freestanding Si_3_P_4_ NPs with cubic crystal structure was successfully carried out and corroborated by XRD, TEM, EDX, electron diffraction, and Raman spectroscopy. The cell parameter of the experimentally obtained Si_3_P_4_ is 5.04 Å. The formation of the considered substance in the form of polycrystalline NPs occurs at 400 °C (further heating causes progressive decomposition). Vibrational spectra of a 3:4 binary compound with defective zinc blende structure were recorded and analyzed for the first time. Several techniques for functionalization and colloidal solubilization of Si_3_P_4_ NPs were outlined. Optical properties of the material were studied to reveal an experimental bandgap of 1.25 eV. Potential applications of the material include the preparation of microcircuits through the introduction of P through diffusion doping of wafers. Incorporating a gaseous etching step into the annealing process widens the applicable use to syntheses of novel nanosized silicon selenides, arsenides, and sulfides.

## Methods

Distilled water, acetonitrile (reagent grade), 1-dodecanol (analytical grade), and hexane (reagent grade) were used for sol preparation; 40% hydrofluoric acid (pure) was used for etching. Si NPs were synthesized by laser-induced chemical vapor deposition using a silane precursor (the average particle diameter was 20 nm [41]). The NPs oxidized when stored in air, and the resultant mass fraction of Si was 77%.

Hydrogenated Si NPs were obtained via gaseous etching of Si NPs. The process was conducted in an air-tight polytetrafluoroethylene chamber connected with a vacuum pump and a separate reservoir with 0.5 mL of HF with a three-position valve (to address the highly toxic and corrosive nature of HF). The total etching time was 20 min (HF vapor etching of Si NPs doped with P results in the removal of the oxide layer after 15 min of etching [42]). It should be mentioned that Si NPs with hydrogen-terminated surfaces rapidly oxidize when exposed to air even at room temperatures [43–44] (the benefit of the gaseous etching is that the particles remain dry, resulting in a lower oxidation rate [45]). One of the samples had also been pre-annealed in vacuum at 900 °C for 6 h before the etching step to estimate the influence of an amorphous surface layer of the NPs on Si_3_P_4_ phase formation.

In order to obtain silicon phosphide samples, weighted amounts of hydrogenated Si NPs (ca. 10 mg) and red phosphorus (ca. 15 mg) were transferred to a clean degassed quartz ampoule, which was sealed under the residual pressure of 5 × 10^−2^ mbar. Annealings were carried out in a muffle furnace SNOL-3/11 (Technotherm); the annealing conditions are given in Table 1.

The duration of each experiment correlated with the rate of reaction depending on the synthesis temperature. Residual phosphorus was transferred to the end of the ampoule with a temperature gradient after the synthesis. In an experiment where a sample was obtained after annealing at 400 °C, hydrosylilation was performed upon sealing off excess phosphorus at the end of the ampoule.

Prior to recording IR spectra of hydrogenated Si NPs on a Frontier infrared Fourier spectrometer (Perkin Elmer), the NPs were dispersed in hexane by sonication in an ultrasonic bath SW3H (Sono Swiss). Once the films were drop cast on aluminum mirrors, the IR spectra were recorded.

The synthesized Si_3_P_4_ NPs were reactive and emanated phosphine upon contact with moisture. Once the ampoules were opened in an inert atmosphere, particles were allowed to gradually form on the surface oxide layer to prevent combustion. In an attempt to prevent hydrolysis/oxidation, a quartz ampoule with NPs synthesized at 400 °C was opened inside an evacuated and sealed copper ampoule partially filled with octadecene. Hydrosilylation was carried out for 30 min at 315 °C. In the case of alcoholysis passivation, degassed 1-dodecanol was introduced to the Si_3_P_4_ product powder without air contact.

Si_3_P_4_ NPs samples were examined on a diffractometer DRON-4-07 (Cu Kα radiation) in the form of films on a polished quartz substrate; phase analysis was performed using the program “WinXPow”. Elemental analysis was carried out on an X-ray fluorescence spectrometer S2 Picofox (Bruker) with total external reflection. Transmission electron microscopy (TEM) samples were dispersed in an ultrasonic bath with hexane and then deposited on copper grids with lacey carbon film (SPI Supplies, USA). TEM was carried out on a FEI Osiris microscope (Thermo Fisher Scientific, USA) at an accelerating voltage of 200 kV.

Raman scattering of pellets of Si_3_P_4_ samples was obtained with an iRamanPlus (BW Tech) portable Raman spectrometer (532 nm laser). The integration time was 10 min, and eight acquisitions were averaged to obtain each spectrum.

A Cary 50 (Varian) spectrophotometer was used for the study of optical absorption. The sample was examined in the form of acetonitrile sol in a quartz SUPRASIL cuvette with an optical path length of 1 cm. Spectra were registered in the wavelength range of 190–1100 nm. The mass concentration of Si_3_P_4_ material in the sol (for the sake of attenuation coefficient calculation) was determined by drop casting 3.00 μL of the sol onto a sapphire substrate followed by an XRF study of the resultant film.

DFT-GGA computations (structure optimization and calculation of vibrational frequencies) were performed with the Quantum ESPRESSO package [46–47] on a 9 × 9 × 9 Monkhorst–Pack *k*-point grid. The functional of choice was PBE with Grimme's DFT-D2 dispersion correction [48]; ultrasoft RRJK pseudopotentials were used [49]. Cutoff energies for plane waves were set at 60 Ry, and for charge density at 240 Ry.

## Supporting Information

File 1 Additional experimental data.
