# Supplementary material for: Isolation of cubic Si3P4 in the form of nanocrystals
Source: Beilstein J Nanotechnol. 2023 Sep 26;14:971–9. doi: 10.3762/bjnano.14.80 (PMC10548250; doi:10.3762/bjnano.14.80)
Supplement: File 1 — Additional experimental data. [file Beilstein_J_Nanotechnol-14-971-s001.pdf]

BEILSTEIN JOURNAL OF NANOTECHNOLOGY

Supporting Information

for

Isolation of cubic Si_3P_4 in the form of nanocrystals

Polina K. Nikiforova, Sergei S. Bubenov, Vadim B. Platonov, Andrey S. Kumskov, Nikolay N. Kononov, Tatyana A. Kuznetsova and Sergey G. Dorofeev

Beilstein J. Nanotechnol. **2023,** *14,* 971–979. doi:10.3762/bjnano.14.80

Additional experimental data

License and Terms: This is a supporting information file under the terms of the Creative Commons Attribution License (https://creativecommons.org/licenses/by/4.0). Please note that the reuse, redistribution and reproduction in particular requires that the author(s) and source are credited and that individual graphics may be subject to special legal provisions.
The license is subject to the *Beilstein Journal of Nanotechnology* terms and conditions: (https://www.beilstein-journals.org/bjnano/terms)

Selected temperature for doping of Si NPs with phosphorus and the calculation of the diffusion constant resulted from the equation $D = D_0 e^{\frac{-Q}{RT}}$, where D_0=20 cm^2/sec, Q=364 kJ/mol, T=670°C [1].

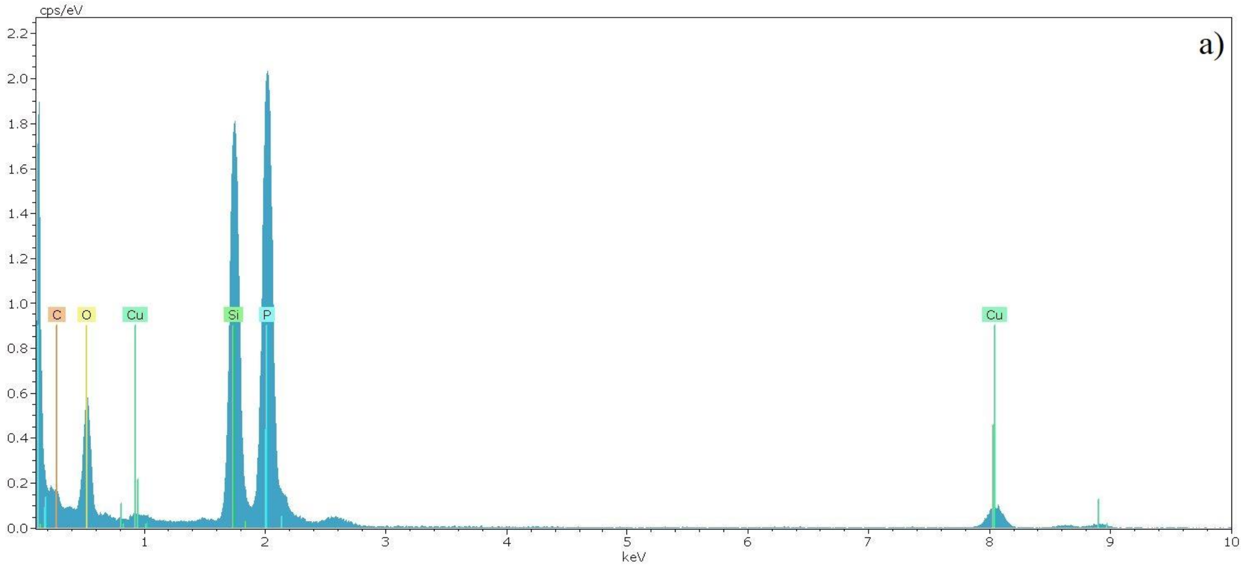

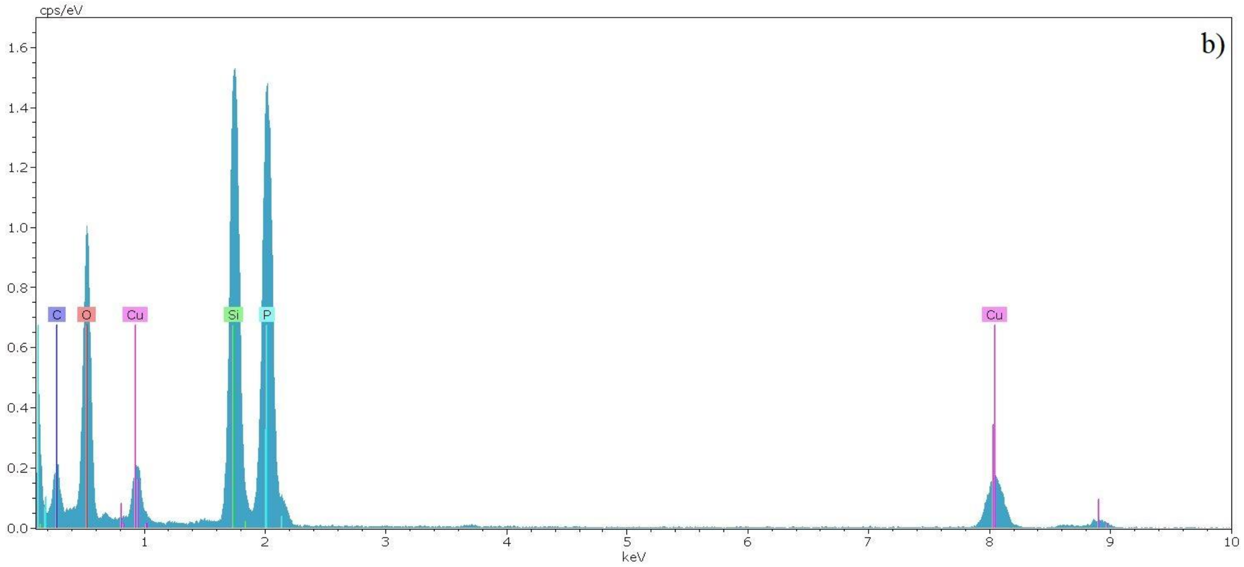

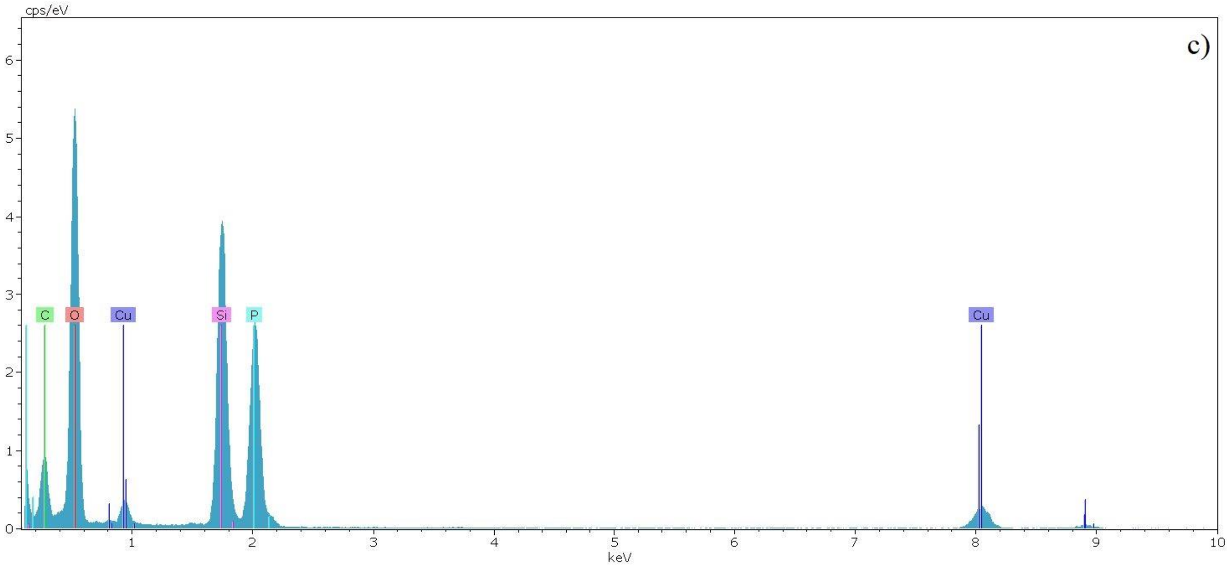

Figure S1: EDX spectra of (a),(b) – SP550, (c) – SP670.

Table S1: The correlation of bands and wavenumbers for Si NPs, hydrogenated Si NPs and SP400, SP670 and SP900 samples.

Assignment	Wavenumber, cm^{-1}					
	Reference data	Sample				
		Si NPs	Hydrogenated Si NPs	SP400	SP670	SP900
O-H stretching	3700-3200 [2]	3420	3509 3450	-	3450	3329
=C-H stretching	3100-3000 [2]	-	-	3073	-	-
Methyl asymmetric C-H stretching	2960 [3]	2960	2957	2956	2960	2956
Methylene asymmetric C-H stretching	2930 [2]	2925	2924	2925	2926	2921
Methylene symmetric C-H stretching	2850 [2]	2854	2855	2853	2857	2853
(O_3)Si-H stretching	2250 [4]	2267	-	-	-	2266
(Si_3)Si-H stretching	2100 [4]	2105	2107	-	-	-
Overtone $HRC=CH_2$	1850-1800 [3]			1821		
C=O stretching	1730 [2]	1728	1711	1743-1730-1718	1714	1720
H_2O deformation	1640 [5]	1623	1631	-	1646	1641
C=C stretching	1680-1600 [2]	-	-	1642	-	-
Methylene scissoring	1465 [2]	1465	1466	1466	1469	1465
Methyl symmetrical C-H bending	1380 [2]	-	-	1378	-	-

Methylene twisting	1300 [2]	-	-	1302	-	-
Si-O-Si stretching	1200-1000 [2], [6]	1208 1069	1167 1072	1186 1098	1205 1090	1183 1073
C-H deformation non-planar (vinyl)	995-985 [3]	-	-	992	-	-
C-H deformation non-planar (vinyl)	915-905 [3]	-	-	911	-	-
H-Si-O_3 deformation	880 [6]	880	-	-	-	874
H-Si-O_2 deformation	850 [6]	-	863	-	-	-
Si-O-Si symmetrical stretching	810 [7]	804	814	806	801	806
Methylene rocking	720 [2]	-	720	721	-	-
=C-H out-of-plane bending	1000-600 [2]	-	-	668	-	-
Si-Si-H_x deformation	637 [6]	633	-	-	-	-
Si-P stretching	-	-	-	494	492	490
Si-O-Si rocking	450 [7]	452	-	-	-	-

REFERENCES

[1] Babichev, A.P.; Babushkina, N.A.; Bratkovsky, A.M. Physical quantities. In *Diffusion*; Grigoriev, I.S.; Meilikhov, E.Z., Eds.; Energoatomizdat: Moscow, 1991; pp 375–390.

[2] Stuart, B. H. *Infrared Spectroscopy: Fundamentals and Applications*; John Wiley & Sons: Hoboken, N.J., 2004.

[3] Tarasevich, B. N. *IR Spectra of Main Classes of Organic Compounds*; Moscow State University: Moscow, 2012.

[4] Matsumoto, K.; Inada, M.; Umezu, I.; Sugimura, A. *Jpn. J. Appl. Phys.* **2005,** *44,* No. 12, 8742–8746.

[5] Mendelovici, E.; Villalba, R.; Sagarzazu, A.; Carias, O. *Clay Miner.* **1995,** *30,* 307–313.

[6] Zhou, S.; Pi, X.; Ni, Z.; Ding, Y.; Jiang, Y.; Jin, C.; Delerue, C.; Yang, D.; Nozaki, T. *ACS Nano* **2015**, *9,* No. 1, 378–386.

[7] Gunde, M.K. *Physica B* **2000,** *292***,** 286–295.
